# *ITGA4* gene methylation status in chronic lymphocytic leukemia

**DOI:** 10.2144/fsoa-2020-0034

**Published:** 2020-06-26

**Authors:** Hanaa RM Attia, Mona Hamed Ibrahim, Shereen H Abd El-Aziz, Naglaa M Hassan, Randa A Osman, Heba A Hagag, Marianne E Yassa, Amany H Abdelrahman, Iman I Salama, Mohamed Emam Sobeih

**Affiliations:** 1 Clinical & Chemical Pathology Department, Medical Division, National Research Centre, Centre of Excellence, Cairo, Egypt; 2 Clinical Pathology Department, National Cancer Institute, Cairo University, Cairo, Egypt; 3 Clinical Pathology Department, Flow Cytometry Unit, National Cancer Institute, Cairo University, Cairo, Egypt; 4 Cytogenetic Unit – Main Lab., Kasr Al-Ainy School of Medicine, Cairo University, Cairo, Egypt; 5 Clinical & Chemical Pathology Department, Kasr Al-Ainy School of Medicine, Cairo University, Cairo, Egypt; 6 Clinical & Chemical Pathology Department, National Research Centre, Cairo, Egypt; 7 Community Medicine Research Department, National Research Centre, Cairo, Egypt; 8 Medical Oncology Department, National Cancer Institute, Cairo University, Cairo, Egypt

**Keywords:** beta 2 microglobulin, chronic lymphocytic leukemia, cytogenetics, *ITGA4* gene expression, methylation analysis, prognostic markers, pyrosequencing

## Abstract

**Background:**

We aimed to investigate *ITGA4* gene expression pattern and to explore its methylation heterogeneity in chronic lymphocytic leukemia (CLL).

**Patients & methods:**

Eighty one CLL patients and 75 healthy subjects were enrolled and prognostic evaluation of patients was assessed. *ITGA4* q-realtime PCR was performed using Applied Biosystems, TaqMan gene expression assay. *ITGA4* gene-specific CpG methylation was investigated in real time using pyrosequencing technology.

**Results:**

*ITGA4* was differentially expressed in CLL patients. The CpG sites-1, 2 and 3 showed significantly higher mean levels than healthy controls (p = <0.001, 0.007 and 0.009). Significant association between CpG site-1 and CLL has been detected using age-adjusted logistic regression (p < 0.001).

**Conclusion:**

Hypermethylation at *ITGA4* gene CpG sites (1,2,3) is a characteristic feature in CLL.

## Lay abstract

Chronic lymphocytic leukemia (CLL) is a clonal lymphoproliferative disorder that is characterized by heterogeneous presentation. The *ITGA4* gene (*CD49d*) encodes a member of the integrin alpha chain family of proteins. With this study we aimed to investigate the *ITGA4* gene expression by q-realtime PCR and to explore its methylation heterogeneity using pyrosequencing technology in CLL. We found that the *ITGA4* gene was differentially expressed in CLL patients. A significant association between CpG site-1 and CLL has been detected using age-adjusted logistic regression analysis. Hypermethylation at *ITGA4* gene CpG sites (1,2,3) is a characteristic feature in CLL when compared with healthy controls.

Chronic lymphocytic leukemia (CLL) is a clonal lymphoproliferative disorder that is characterized by heterogeneous presentation at the clinical and molecular levels. Many investigations into CLL genetics have provided more understanding of CLL biology and a plethora of new prognostic markers have been delivered for predicting patient outcomes and response to chemotherapy or targeted therapy [1–3]. ITGA4 protein has been found to be deregulated in CLL with adverse clinical outcome. *ITGA4* gene (*CD49d*) encodes a member of the integrin alpha chain family of proteins and is considered a negative prognosticator in CLL with aggressive course and short time to treatment [4–7]. It is implicated in B-cell binding to the microenvironment and stromal cells found in the germinal centers of lymphoid follicle. It also serves as a signaling receptor that upregulates BCL-2 transcription, which in turn delivers prosurvival signals, inhibits apoptosis and protects CLL cells from drug-induced damages [8,9]. DNA methylation is considered a new approach in CLL genetics to provide diagnostic and prognostic biomarkers for beneficial use in clinical settings. The CLL epigenome shows global hypomethylation and local hypermethylation with increased opportunities for somatic mutations [10–12]. The interplay between hypo- and hyper-methylation in the CLL genome highlighted the methylation-dependent role in gene expression deregulation and its impact on malignant transformation and disease progression [13]. *ITGA4* overexpression in trisomy 12 CLL patients was proved to be regulated by a methylation dependent mechanism [5]. Altered methylation patterns of promotor regions in the genes have been detected previously and proposed as prognostic markers in CLL [14,15]. Kulis *et al.* reported that CLL patients with mutated or unmutated IGHV have different epigenetic signature from normal B cell subpopulations using whole-genome bisulfite sequencing and high-density microarrays [15]. In this study we aimed to investigate *ITGA4* gene expression pattern and to explore its methylation heterogeneity in CLL. Also, we aimed to identify their association with different well-established prognostic variables that can guide management strategies and targeted therapy.

## Patients & methods

Eighty one CLL patients and 75 healthy controls were enrolled for the study between 2017 and 2019. Patients were diagnosed according to WHO classification of hemato-lymphoid tumors [16]. The study was approved by ethical committee of National Research Centre (registration number 17-147). All participants gave informed consent before being included in accordance with the code of ethics of the World Medical Association (Declaration of Helsinki) for experiments on humans. Fresh peripheral venous blood samples were withdrawn from patients and controls.

For prognostic evaluation, B2M was analyzed using enzyme-linked immunoassay and CD49d, CD38 and cytoplasmic ZAP-70 were assessed as previously described using Beckman Coulter’s Navios Flow Cytometer [17]. The Vysis CLL FISH Probe Kit (List No. 04N02-021, Abbott Molecular, IL, USA) was used to study chromosomes 11, 12, 13 and 17 to determine deletion status of the locus-specific identifier *TP53*, *ATM* and D13S319 probe targets and gain of the D12Z3 sequence in fresh heparinized peripheral blood specimens from patients according to manufacturer’s instructions [18].

### *ITGA4* gene expression & methylation analysis

Total RNA and genomic DNA were extracted from the fresh EDTA blood samples of each subject of both patients and controls promptly after specimen collection with the RNeasy Mini Kit and a QIAamp DNA Mini Kit (QIAGEN, Strasse 1 40724 Hilden, Germany), respectively according to the manufacturer’s guidelines using automated QiaCube^®^ nucleic acid extractor (QIAGEN). Integrity and quality of the purified RNA and DNA were measured initially in duplicates by NanoDrop 2000c spectrophotometer^®^ (Thermo Fisher Scientific Inc., DE, USA); verified on Qubit^®^ 2.0 fluorometer (Invitrogen, Thermo Fisher Scientific Inc., [Life Technologies Holdings Pte Ltd, MA, USA]) using high sensitivity dsDNA and RNA quantitation Kits.

#### *ITGA4* gene expression

High-capacity cDNA Reverse Transcription Kit (Applied Biosystems, Thermo Fisher Scientific Inc., Vilnius, Lithuania) was employed for c-DNA synthesis according to the manufacturer’s guidelines in a total reaction volume of 20 ul with the following thermal profile: 25°C for 10 min; 37°C for 120 min; 85°C for 5 min; finally 4°C indefinitely.

*ITGA4* gene expression by q-realtime PCR was performed using TaqMan Gene Expression assay, (ID: Hs00168433_m1, cat. no. 4331182), (Applied Biosystems, Thermo Fisher Scientific Inc.) on the QuantStudio 12K flex Real-Time PCR system (Applied Biosystems-Life Technologies, CA, USA). Experiment setup profile was as follows; hold at 50°C for 2 min, hold at 95°C for 10 min, then PCR stage (40 cycles): denature at 95°C for 15 s then anneal/extend at 60°C for 1 min. The expressions were quantitated relative to the *B2M* housekeeping gene (ID: Hs00187842_m1) using the 2ΔΔCT method to calculate the relative expression of *ITGA4* gene in the studied samples.

#### Methylation of the *ITGA4* gene (ENSG00000115232, chromosome location chr2. q31.3:182,321,575–182,323,696) with four intronic CpG sites

Genomic DNA was sodium bisulfite modified to selectively convert cytosine to uracil using the QIAGEN EpiTect Fast DNA Bisulfite Kit (cat no. 59824). Bisulfite conversion thermal cycling conditions were as follows: denaturation for 5 min at 95°C; incubation for 10 min at 60°C; hold indefinitely at 20°C. Cleanup of converted DNA was done according to the manufacturer’s protocol.

PCR was performed using PyroMark PCR kit from QIAGEN (cat.no: 978703), including primers designed specifically to bisulfite-modified DNA, one of them is biotinylated primer at its 5′ end for pyrosequencing template preparation. Cycling protocol was as follows: initial activation for 15 min at 95°C; three step cycling for 45 cycles: denaturation for 30 s at 94°C; annealing for 30 s at 56°C; extension for 30 s at 72°C then final extension for 10 min at 72°C. Then agarose gel verification of amplicon (121 bp) was performed to verify that we have a single, sharp band and no unincorporated primers.

*Pyrosequencing analysis*: *ITGA4* CpG assay Kit (Hs_ITGA4_01_PM PyroMark CpG assay, cat no. PM00097489, QIAGEN) was used for *ITGA4* gene-specific CpG methylation in real time using pyrosequencing technology on the PyroMark Q24 System with software v.2.0. The kit includes 10× PCR primer set and 10× sequencing primers. Four CpG dinucleotide pairs (sites) were analyzed.

Analyzed sequence was: CGGCGTGAGAATGGCGCCCTAGGGATTCCCTGCCCGA and the nucleotide dispensation order was set according to the manufacturer’s instructions: GTCGTCGATGAGATAGTCGTTAGAGTTGTCG.

### Statistical methods

Data were coded and entered using the statistical package for the Social Sciences (SPSS) version 25 (IBM Corp., NY, USA). Data were summarized using mean, standard error of the mean, minimum and maximum in quantitative data and using frequency (count) and relative frequency (percentage) for categorical data. Comparisons between quantitative variables were done using t-test and for the nonparametric data Kruskal–Wallis and Mann–Whitney tests were used [19]. One sample Wilcoxon signed rank test was used to test for gene fold change significance. For comparing categorical data, Chi square (χ^2^) test was performed. Exact test was used instead when the expected frequency is less than 5 [20]. Correlations between quantitative variables were done using Spearman correlation coefficient. p-values less than 0.05 were considered as statistically significant.

## Results

Demographic, clinical and laboratory findings of CLL patients and controls are presented in Table 1. *ITGA4* was differentially expressed in CLL patients with mean fold change of 1.463 ± 0.11 and p < 0.001. No statistical difference in the *ITGA4* gene expression mean fold change has been observed in patients with different Binet stage or disease course whether indolent or aggressive (p = 0.417 and 0.319, respectively). CLL patients were categorized according to *ITGA4* fold change into two groups. Upregulated expression of *ITGA4* more than 1.5-fold change was detected in 46.9% of all patients. A subset of CLL patients presented with low expression of *ITGA4* gene (24.69%). Both groups were age and sex matched. Laboratory variables in different patient categories based on *ITGA4* fold change are demonstrated in Table 2. Lymphadenopathy was detected in 72.8% of CLL patients and observed more in the high *ITGA4* expression subset of CLL patients with p = 0.026. Higher mean expression percentage of ITGA4 protein by flowcytometry and trisomy12 were observed in patients with upregulated gene when compared with patients with downregulated gene (p = 0.001 and 0.046, respectively). No significant difference was detected in the mean levels of expression percentage of CD38, ZAP-70, del17p, del11q and del13q between both upregulated and downregulated gene groups.

**Table 1.** Demographic, clinical and laboratory findings of chronic lymphocytic leukemia patients and controls.

Character	CLL patients (n = 81)	Controls (n = 75)	p-value
Gender – Males, n (%) – Females, n (%)	47 (58.02) 34 (41.98)	46 (61.33) 29 (38.67)	0.362 (NS)
Age (years), mean ± standard deviation	58.25 ± 9.67	51.18 ± 7.0	0.125 (NS)
Course of disease: – Newly diagnosed, n (%) – Indolent, n (%) – Aggressive, n (%)	0 (12.35) 32 (39.51) 39 (48.14)	–	–
Staging (Binet) at diagnosis, – Stage A, n (%) – Stage B, n (%) – Stage C, n (%)	11 (13.6) 29 (35.8) 41 (50.6)	–	–
Family history of leukemia or other types of cancer, n (%)	14 (17.3)	6 (8)	0.000^‡^
Smoking, n (%)	24 (29.6)	15 (20)	0.073 (NS)
Clinical findings: – Lymphadenopathy, n (%) – Splenomegaly, n (%) – Hepatomegaly, n (%)	59 (72.8) 56 (69.1) 34 (41.9)	–	
*ITGA4* gene regulation – Downregulated <0.5-fold – Upregulated >1.5-fold	20 (24.7) 38 (46.9)	3 (4.0) 29 (38.7)	0.034^†^
*ITGA4* gene methylation % – CpG site-1 % (mean ± SE) – CpG site-2 % (mean ± SE) – CpG site-3 % (mean ± SE) – CpG site-4 % (mean ± SE)	26.9 ± 0.93 2.9 ± 0.16 3.7 ± 0.19 7.6 ± 0.28	7.4 ± 0.49 2.1 ± 0.2 2.9 ± 0.0.2 6.9 ± 0.26	<0.001^‡^ 0.007^‡^ 0.009^‡^ 0.149

† p < 0.05 is considered significant.

‡ p < 0.01 is considered highly significant.

CLL: Chronic lymphocytic leukemia; NS: Non-significant; SE: Standard error.

**Table 2.** Comparison between demographic and laboratory variables in different *ITGA4* fold change categories of chronic lymphocytic leukemia patients.

Variables	*ITGA4* <0.5-fold change CLL patients (n = 20)	*ITGA4* >1.5-fold change CLL patients (n = 38)	p-value
Gender – Males, n (%) – Females, n (%)	14 (70) 6 (30)	20 (52.6) 18 (47.4)	0.250 (NS)
Age (years), (mean ± SE)	58.2 ± 2.9	57.3 ± 1.7	0.797 (NS)
B2M (µg/ml), (mean ± SE)	4.7 ± 0.69	5.8 ± 0.55	0.268 (NS)
ITGA4 protein expression %(mean ± SE)	4.4 ± 2.3	20.8 ± 4.8	0.001^‡^
CD38 % (mean ± SE)	21.6 ± 7.3	13.9 ± 2.7	0.334 (NS)
ZAP-70 % (mean ± SE)	1.9 ± 1.3	5.1 ± 2.2	0.353 (NS)
Trisomy12 % (mean ± SE)	2.14 ± 0.376	9.08 ± 3.3	0.046^†^
Del17p % (mean ± SE)	3.14 ± 0.619	2.58 ± 0.3	0.358 (NS)
Del11q % (mean ± SE)	16.86 ± 8.5	7.0 ± 3.3	0.297 (NS)
Del13q14 % (mean ± SE)	31.7 ± 9.3	7.9 ± 3.7	0.079 (NS)
*ITGA4* gene methylation			
– CpG site-1 % (mean ± SE)	31.1 ± 2.0	25.4 ± 1.4	0.029^†^
– CpG site-2 % (mean ± SE)	3.9 ± 0.5	2.5 ± 0.157	0.01^†^
– CpG site-3 % (mean ± SE)	4.3 ± 0.57	3.4 ± 0.25	0.088 (NS)
– CpG site-4 % (mean ± SE)	8.1 ± 0.76	7.3 ± 0.3200	0.347 (NS)

† p < 0.05 is considered significant.

‡ p < 0.01 is considered highly significant.

CLL: Chronic lymphocytic leukemia; SE: Standard error; NS: Non-significant.

**Figure 1.**
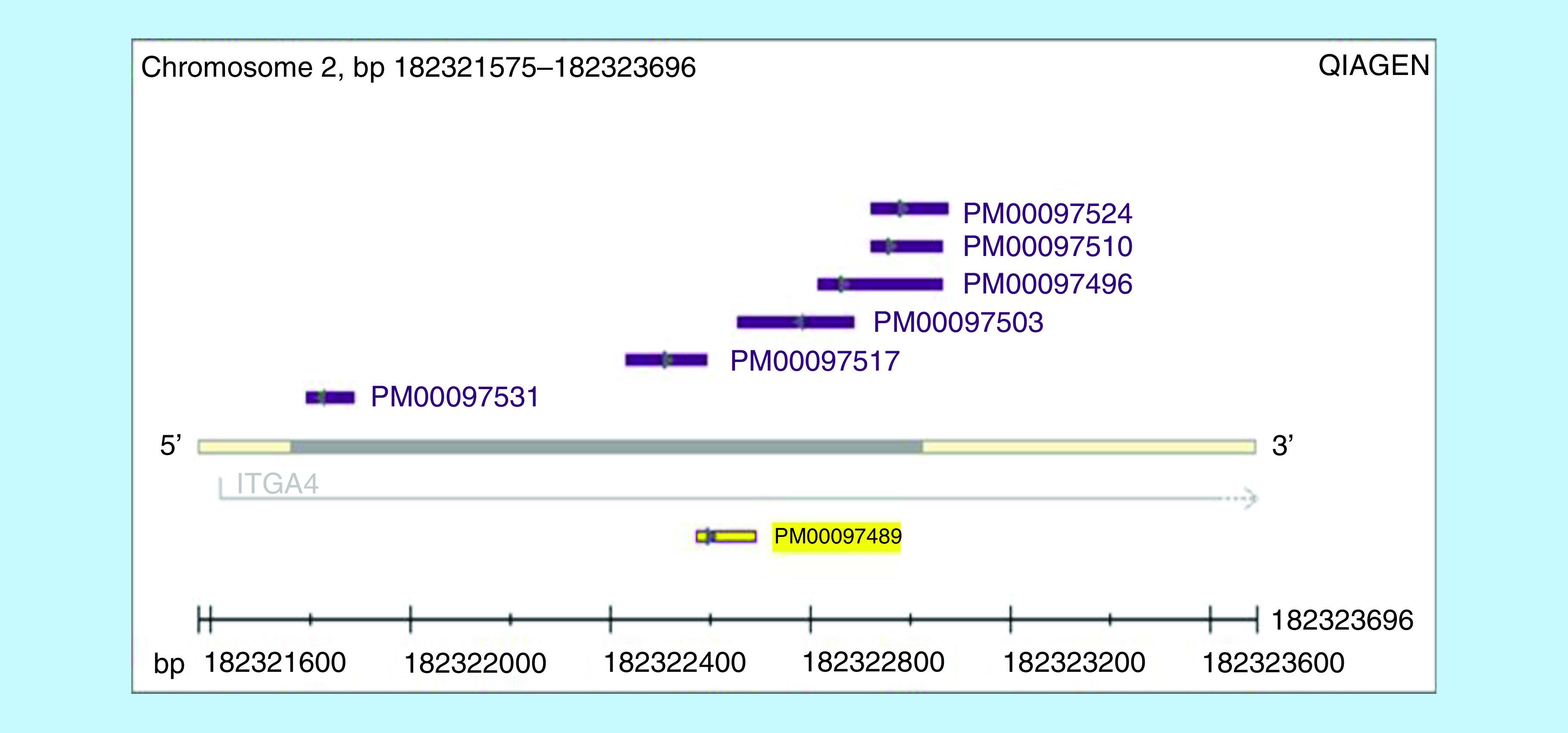
ITGA4; genomic location. *ITGA4* gene specification and chromosome location according to GeneGlobe specification, [21].

**Figure 2.**
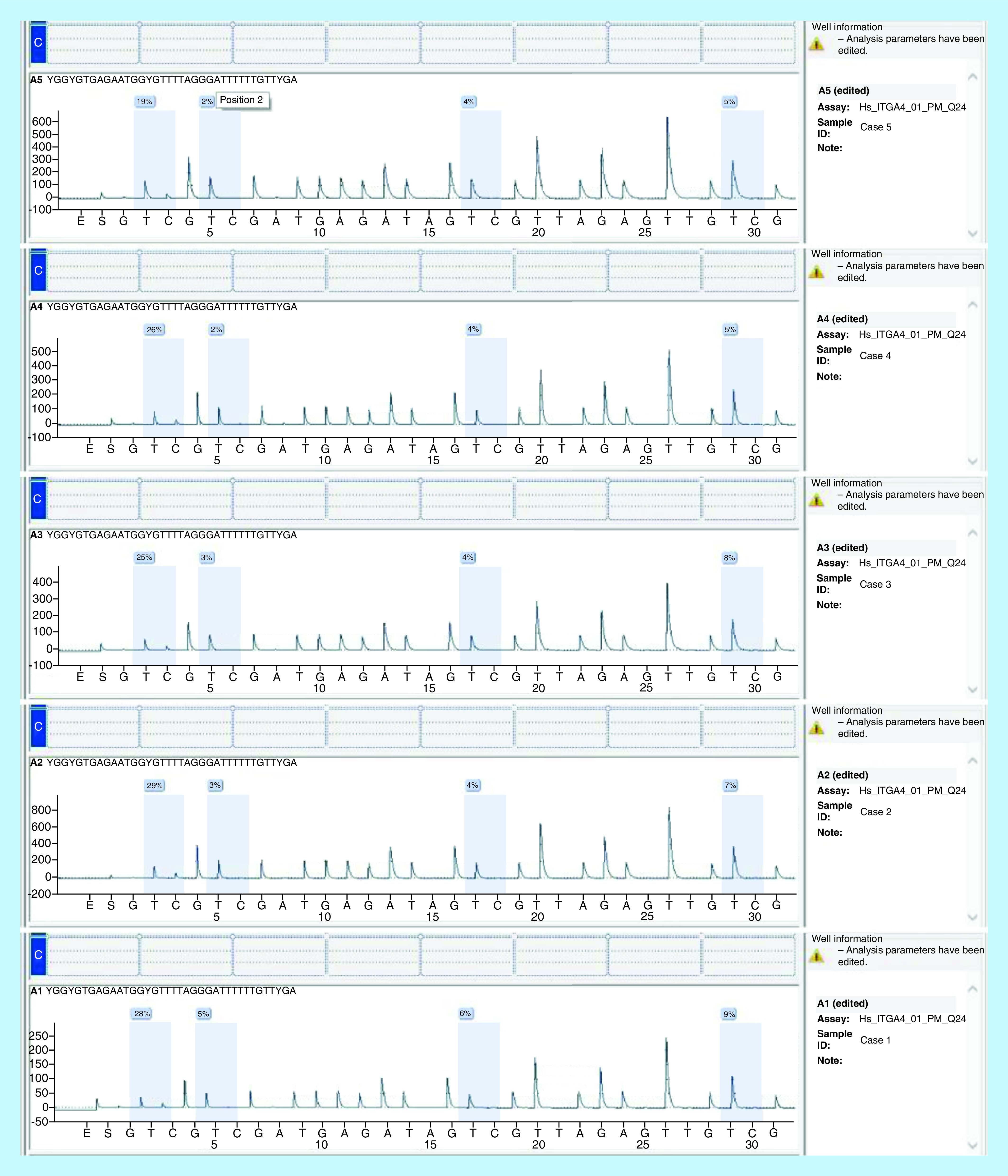
*ITGA4* gene methylation (%) of the four CpG sites by pyrosequencing of some chronic lymphocytic leukemia patients.

**Figure 3.**
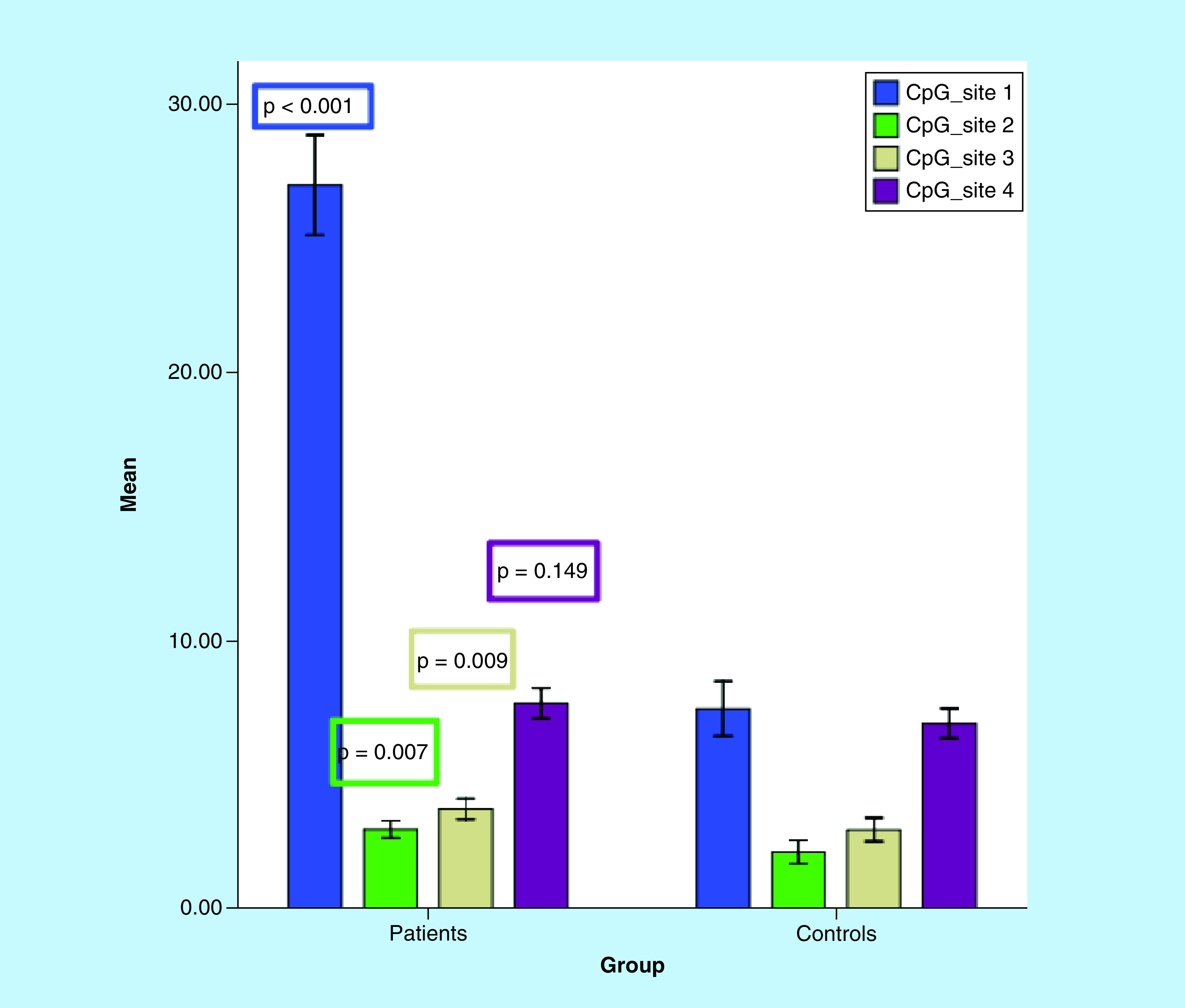
Comparison between *ITGA4* gene methylation (%) of the four CpG sites by pyrosequencing between chronic lymphocytic leukemia patients (n = 81) and controls (n = 75). The CpG sites-1,2,3 showed significantly higher mean levels in chronic lymphocytic leukemia patients than healthy controls (p = <0.001, 0.007 and 0.009, respectively). Error bars: 95% CI.

Methylation analysis of *ITGA4* gene (four intronic CpG sites) was performed with GeneGlobe specification, presented in Figure 1 [21]. Methylation pyrogram of some CLL patients is shown in Figure 2. Comparison between *ITGA4* gene methylation (%) of the four CpG sites between CLL patients and controls is demonstrated in Table 1 & Figure 3. The CpG sites-1, 2 and 3 showed significantly higher mean levels in CLL patients than healthy controls (p < 0.001, 0.007 and 0.009, respectively). By comparing the mean methylation % of the four CpG sites in different patient categories based on *ITGA4* gene expression fold change, CpG sites-1 and 2 showed significantly higher mean methylation % in patients with downregulated gene than patients with upregulated gene, (p = 0.029 and 0.01, respectively) as shown in Table 2.

**Figure 4.**
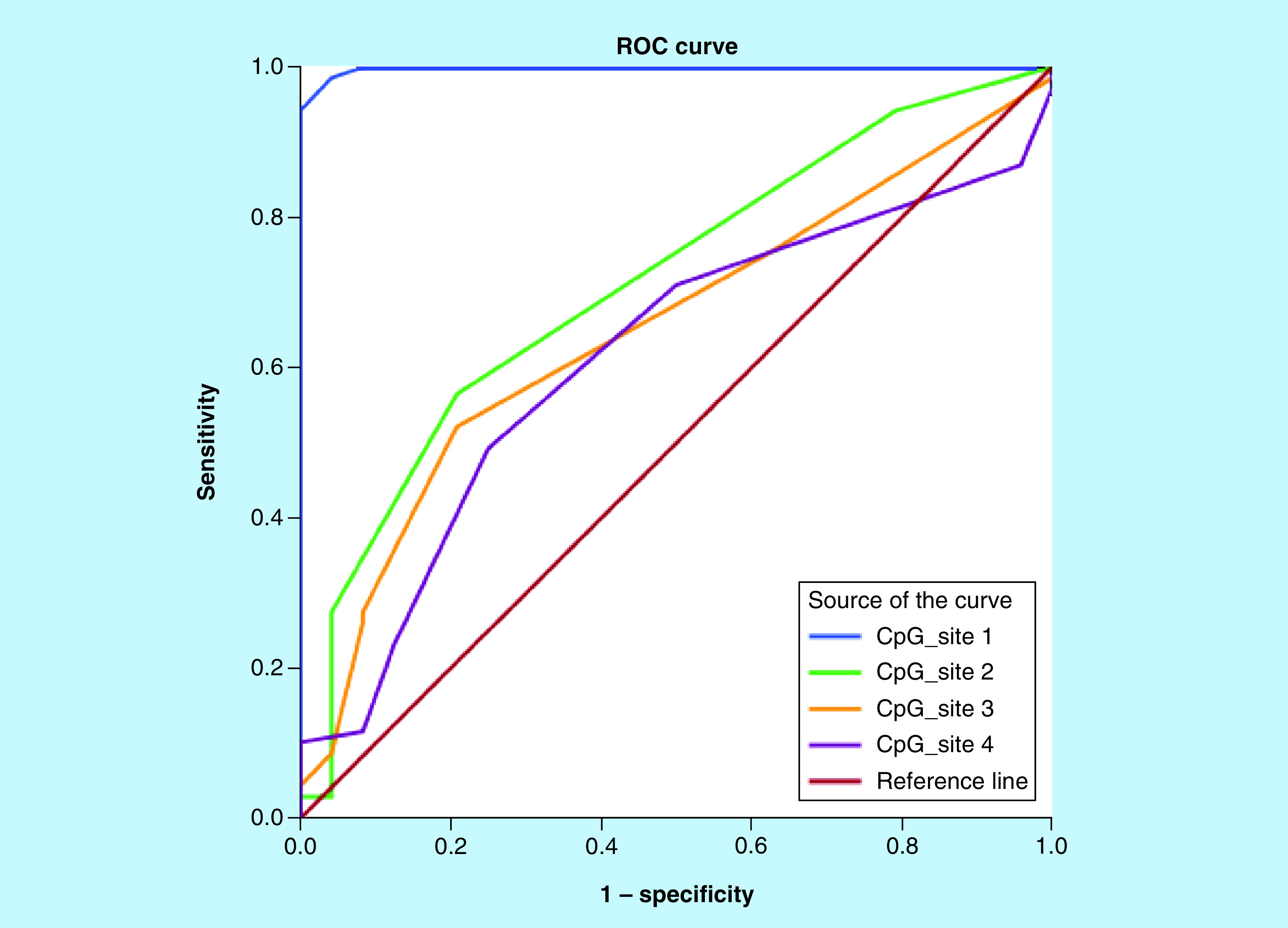
Receiver operating characteristics curves for the ITGA4 methylation % of the four CpG sites among patients and healthy controls. ROC: Receiver operating characteristics.

Receiver operating characteristic analysis was done for the four CpG sites to discriminate between CLL patients and healthy controls (Figure 4). Cut-off values were established for CLL diagnostic and prognostic evaluation (Table 3). The frequency of *ITGA4* hypermethylation of the significant CpG sites among patients and controls is presented in Table 4.

**Table 3.** Cut-off values of *ITGA4* methylation % of the four CpG sites for chronic lymphocytic leukemia diagnostic and prognostic evaluation.

Variables	Cutoff %	Sensitivity %	Specificity %	AUC	95% CI	p-value
CpG site-1	12.5	98.6	95.8	0.998	0.994–1.002	<0.001^‡^
CpG site-2	2.5	56.5	79.2	0.713	0.597–0.829	0.002^‡^
CpG site-3	3.5	52.2	79.2	0.652	0.532–0.771	0.028^†^
CpG site-4	7.5	49.3	75.0	0.612	0.488–0.736	0.102

† p < 0.05 is considered significant.

‡ p < 0.01 is considered highly significant.

AUC: Area under the curve.

**Table 4.** Frequency of *ITGA4* hypermethylation of the significant CpG sites among patients and controls.

*ITGA4* gene methylation % of CpG sites	CLL patients, number (%)	Controls, number (%)	Odds ratio (95% CI)	Chi-square significance (p-value)
CpG site-1 >12.5 %	80 (98.7)	3 (4)	23.6 (3.47–161.1)	<0.001^†^
CpG site-2 >2.5 %	46 (56.8)	16 (21.3)	1.448 (1.131–1.853)	0.004^‡^
CpG site-3 >3.5 %	42 (51.8)	16 (21.3)	1.384 (1.093–1.751)	0.009^‡^

† p < 0.05 is considered significant;

‡ p < 0.01 is considered highly significant.

CLL: Chronic lymphocytic leukemia.

**Figure 5.**
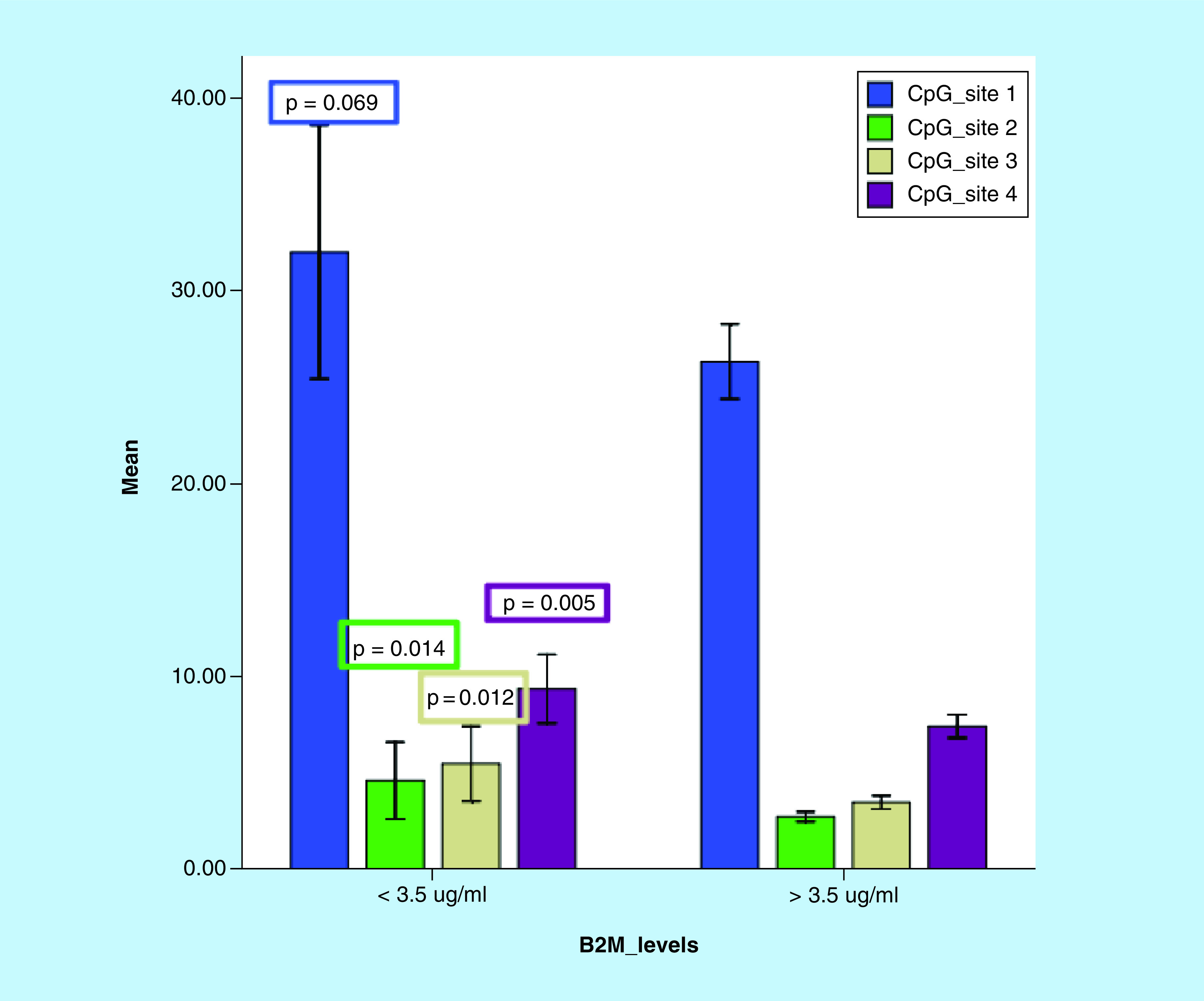
Comparison between *ITGA4* gene methylation (%) of the four CpG sites by pyrosequencing between different groups of chronic lymphocytic leukemia patients based on B2M levels (patients with B2M >3.5 µg/ml, n = 62; patients with B2M <3.5 µg/ml, n = 19). Error bars: 95% CI.

Based on cytogenetics findings, patients were stratified to identify the differences in the *ITGA4* CpG sites. Patients with del13q14+ expression showed significant increase in the mean methylation levels of CpG site-2 (mean ± SE; 4.1 ± 0.57 vs 2.7 ± 0.15; p = 0.015) and CpG site-3 (mean ± SE; 4.6 ± 0.6 vs 3.4 ± 0.2; p = 0.029). Furthermore, patients were categorized according to B2M levels to investigate methylation status of *ITGA4* CpG sites in both high and low levels of B2M and statistical comparison was performed (Table 5 & Figure 5).

**Table 5.** Comparison between *ITGA4* gene methylation % of the four CpG sites among patients with low and high levels of beta 2 microglobulin.

*ITGA4* gene methylation %	CLL patients with low B2M <3.5 µg/ml (n = 9)	CLL patients with high B2M >3.5 µg/ml (n = 72)	p-value
CpG site-1 % (mean ± SE)	32.0 ± 2.7	26.3 ± 0.97	0.069
CpG site-2 % (mean ± SE)	4.6 ± 0.84	2.7 ± 0.12	0.014^†^
CpG site-3 % (mean ± SE)	5.5 ± 0.82	3.49 ± 0.17	0.012^†^
CpG site-4 % (mean ± SE)	9.37 ± 0.75	7.43 ± 0.29	0.005^‡^

† p < 0.05 is considered significant;

‡ p < 0.01 is considered highly significant.

CLL: Chronic lymphocytic leukemia; SE: Standard error.

‘Correlation analysis’ between *ITGA4* gene expression at mRNA level and ITGA4 protein levels defined as mean fluorescent intensity by flow cytometry revealed significant relationship (r = 0.350; p = 0.004). CpG site-1 was correlated significantly to age (r = 0.284; p = 0.041). Age-adjusted logistic regression was performed and significant association between CpG site-1 and CLL has been detected (p < 0.001). Inverse correlations between CpG site-2 and *ITGA4* fold change (r = -0.342, p = 0.004) and between methylation levels at CpG sites-1, 2 and 3 and B2M levels were detected (r = -0.421; p <.001; r = -0.373, p = 0.002; r = -0.349; p = 0.003, respectively). Inverse correlations were observed between methylation levels at CpG sites-1 (r = -0.471; p = 0.01) and CpG site-4 (r = -0.456; p = 0.015) and CD38 expression levels in the subset of patients with upregulated gene.

## Discussion

In the present study, deregulated *ITGA4* gene investigated by q-real-time PCR was more frequently encountered in CLL patients than their healthy counterparts. ITGA4 protein showed higher mean expression by flow cytometry in patients with upregulated gene at mRNA level. Also, a significant correlation has been detected between *ITGA4* gene fold change and ITGA4 protein levels indicating its regulatory role at the level of transcription as previously observed by Zucchetto *et al.* [5]. In patients with upregulated gene, nodal presentation was more frequently observed and mean expression of trisomy12 with intermediate prognostic features was higher than those with downregulation which are consistent with previous flowcytometry based studies [5–7,22].

A subset of our patients showed low expression of *ITGA4* gene which is concordant with previous investigators [23]. Differential gene expression profiles can result from aberrant methylation status that can affect genome stability [13]. To explore the impact of methylation status on heterogeneity in *ITGA4* gene expression, analysis of individual CpG sites in the gene has been investigated and the potential role of their variable levels has been established. The methylation analysis demonstrated that CpG sites-1 and 2 mean levels were elevated in patients with downregulated *ITGA4* gene and inverse correlation was observed between CpG site-2 and *ITGA4* fold change. Furthermore, CLL patients displayed highly significant increase in the means of methylation % of the CpG sites-1, 2 and 3 with more frequent hypermethylation % of the three CpG sites than that detected in their healthy counterparts. Global DNA hypomethylation across the genome has been previously considered a characteristic feature in cancer, whereas DNA hypermethylation was more frequently locally observed [24]. Locally aberrant methylation has been previously described in CLL cells when compared with normal CD19^+^ B cells and has the potential for gene silencing and inactivation of genes related to normal tissue homeostasis [13,25]. Certain introns are considered primary elements directing the gene regulation and causing high gene expression in the genome [26]. So, aberrant methylation of intronic CpG dinucleotides can affect the expression of the gene with possible prognostic impact.

To identify the association between aberrant methylation of the gene with established prognostic markers, patients were categorized based on cytogenetics findings and B2M levels and comparisons have been investigated. High mean levels of CpG sites 2 and 3 were observed in patients with positive expression of del13q14. Furthermore, in the context of high B2M levels, lower mean levels of methylated CpG sites have been observed. Inverse correlations between methylation levels at CpG sites-1, 2 and 3 and B2M levels were detected. These findings postulated a possible favorable impact of hypermethylated *ITGA4* gene identified sites on CLL prognosis. In a previous study, stability of DNA methylation has been reported throughout the course of CLL and in relation to therapy while methylation heterogeneity has been found in more aggressive CLL subgroups [27].

In this study, CpG site-1 was significantly correlated to age. Many investigators suggested that a low level of de novo methylation of CpG islands can take place in normal tissues and increase with age. They postulated that DNA methylation increases with age at some CpG islands and may develop from a small population of cells [28–30]. Age-adjusted logistic regression analysis revealed highly significant association between CpG site 1 and CLL which means that its aberration is related to the disease pathology itself and at a lower level to the aging process.

## Conclusion

In CLL, ITGA4 protein expression is regulated at mRNA level and through local methylation-based mechanism. Methylation status of *ITGA4* gene revealed the significance of measuring CpG site 1 as a prognostic marker for CLL and can be a target for therapy. Hypermethylation at certain identified *ITGA4* gene CpG sites-1, 2 and 3 is a characteristic feature in CLL. CpG sites-2 and 3 mean methylation levels significantly increased in del13q14+ subset of CLL patients. Also CpG sites-1, 2 and 3 were inversely correlated to B2M levels indicating their prognostic impact for further wide scale studies.

## Future perspective

Detailed methylation analysis of different individual CpG sites within the *ITGA4* gene CpG island is recommended to help more understanding of gene regulation in CLL and aiding in future therapeutic trials. Relation to clinical outcome and overall survival with assessment of other strong prognosticators like the IGHV mutational status as well as mutations of TP53, NOTCH1 and SF3B1 are recommended in further studies.

**Summary points**

**Aim**

- In the current study, we aimed to investigate *ITGA4* gene expression pattern and to explore its methylation heterogeneity in chronic lymphocytic leukemia (CLL) that can guide management strategies and targeted therapy.

**Patients & methods**

- Eighty one Egyptian CLL patients and 75 healthy subjects were enrolled for the study.
- CD49d, CD38 and ZAP-70 were assessed using flow cytometer.
- FISH technique was performed for detection of del17p, del11q, del13q14 and trisomy 12.
- B2M was measured by ELISA.
- *ITGA4* gene expression by q-realtime PCR was performed using Applied Biosystems, TaqMan Gene Expression assay and relative quantitation was performed relative to the B2M housekeeping gene.
- *ITGA4* gene-specific CpG methylation in real time using pyrosequencing technology on the PyroMark Q24 System was investigated.

**Results**

- *ITGA4* was differentially expressed in CLL patients (mean fold change of 1.463 ± 0.11 and p < 0.001).
- Upregulated expression was detected in 46.9% of patients. A subset of patients (24.69%) displayed low expression of the gene.
- In methylation analysis, the CpG sites-1,2,3 showed significantly higher mean levels than healthy controls (p = <0.001, 0.007 and 0.009, respectively). CpG sites-1 and 2 showed significantly increased methylation % in patients with downregulated gene, (p = 0.029 and 0.01, respectively).
- Significant association between CpG site-1 and CLL has been detected using age-adjusted logistic regression (p < 0.001).
- Mean methylation levels of CpG sites-2 and 3 significantly increased in del13q14+ subset of CLL patients.
- Inverse correlations between methylation % at CpG sites-1,2,3 and B2M levels were detected.

**Conclusion**

- In CLL, ITGA4 protein expression is regulated at mRNA level and through local methylation-based mechanism.
- Methylation status of *ITGA4* gene revealed the significance of measuring CpG site 1 as a prognostic marker for CLL and can be a target for therapy.
- Hypermethylation at certain identified *ITGA4* gene CpG sites-1, 2 and 3 is a characteristic feature in CLL.
- CpG sites-1, 2 and 3 have prognostic impact for further wide scale studies.
